# Supplementary material for: Polyketide synthase gene clusters in the ericoid mycorrhizal fungus Oidiodendron maius: genome-wide prediction, expression profiling, and functional characterization of knockout mutants
Source: Front Plant Sci. 2026 Sep 16;17:1924430. doi: 10.3389/fpls.2026.1924430 (PMC13623903; doi:10.3389/fpls.2026.1924430)
Supplement: Supplementary file 1 [file SupplementaryFile1.zip › Supplementary Materials/Supplementary Folder 1.zip!/Oidma1_AssemblyScaffolds_Repeatmasked/knownclusterblast/cluster56/jgi.p_Oidma1_138917_mibig_hits.html]

| MIBiG Protein | Description | MIBiG Cluster | MiBiG Product | % ID | % Coverage | BLAST Score | E-value |
| --- | --- | --- | --- | --- | --- | --- | --- |
| AGA37270.1 | 2OG-Fe(II)-oxygenase | BGC0000819 | Paraherquamide\_biosynthetic\_gene\_cluster | 45 | 104.0625 | 308 | 8e-84 |
| AFT91382.1 | EcdK | BGC0000338 | Echinocandin\_B\_biosynthetic\_gene\_cluster | 45 | 103.75 | 290 | 2.3e-78 |
| EPE34350.1 | Clavaminate\_synthase-like\_protein | BGC0001035 | Pneumocandin\_biosynthetic\_gene\_cluster | 42 | 103.75 | 285 | 5.6e-77 |
| AMM63173.1 | AniK | BGC0001371 | Echinocandin\_B\_biosynthetic\_gene\_cluster | 43 | 103.75 | 281 | 1.1e-75 |
| EPE34347.1 | Clavaminate\_synthase-like\_protein | BGC0001035 | Pneumocandin\_biosynthetic\_gene\_cluster | 39 | 102.8125 | 255 | 6.2e-68 |
| AFT91391.1 | HtyE | BGC0000338\_c2 | Echinocandin\_B\_biosynthetic\_gene\_cluster | 39 | 103.125 | 252 | 8.9e-67 |
| AMM63174.1 | AniF | BGC0001371 | Echinocandin\_B\_biosynthetic\_gene\_cluster | 39 | 103.125 | 248 | 7.6e-66 |
| CAM59607.1 | putative\_dioxygenase | BGC0000297 | Aeruginoside\_biosynthetic\_gene\_cluster | 26 | 74.375 | 84 | 2.5e-16 |
